# Strategies for Isolation and Phenotypic, Genetic, and Functional Characterization of Circulating Tumor Cells

**DOI:** 10.3390/cancers12113257

**Published:** 2020-11-04

**Authors:** Maria José Serrano, Jose A Lorente

**Affiliations:** 1 Liquid Biopsy & Cancer Interception Group, GENYO Centre for Genomics and Oncological Research, Pfizer/University of Granada/Andalusian Regional Government, PTS Granada, 18016 Granada, Spain; jlorente@ugr.es; 2 University Hospital Virgen de las Nieves, 18016 Granada, Spain; 3 Department of Pathological Anatomy, Faculty of Medicine, Campus de Ciencias de la Salud, University of Granada, 18016 Granada, Spain; 4 Laboratory of Genetic Identification, Department of Legal Medicine, University of Granada, 18016 Granada, Spain

From the medical and scientific point of view, we are witnessing important changes in the way we approach diseases, and consequently in the way we manage patients [1]. In fact, we live in the era of precision medicine (PM) and personalized medicine (PM), two concepts involving the need to individualize patients’ monitoring as well as their treatment [2]. These new approaches of the health care system respond to a better and greater biological knowledge of diseases such as cancer, thanks to advances in the molecular and cellular biology field. However, these improvements to the molecular and cellular knowledge are also accompanied by technological advances.

Therefore, should the main objective of PM and PP be a more exhaustive and personalized follow-up of patients, it is essential to rely on protocols able to collect samples in an easy, non-invasive way for patients, which are reproducible and highly informative from the clinical and biological point of view. In this context, the concept of liquid biopsy (LB) appears as a complementary or alternative clinical tool to conventional solid biopsy methodologies that are much less reproducible, accessible, expensive and especially aggressive for patients [3]. Despite LB involving analyses of different biomarkers, such as circulating tumor cells (CTCs), circulating tumor nucleic acids (ctNA), microvesicles, platelets or proteins, two of them, CTCs and ctDNA, are currently closer to clinical implementation [4].

In this Special Issue, we wanted to publish and update the most established as well as new technological approaches in the CTCs context. The advances in CTCs technologies are allowing researchers to improve our understanding not only about different phenotypes and genetic diversity but also, and no less importantly, about the functional complexity of CTCs.

This Special Issue, entitled “Special Issue: Strategies for Isolation and Phenotypic, Genetic, and Functional Characterization of Circulating Tumor Cells” is part of a series of Special Issues focused on LB research and its clinical and biological impact in the understanding and analyses of tumoral diseases.

All authors included in this issue are recognized for their special contribution in the CTCs research field. Research was presented both through previous highly cited articles and in the most important conferences. We would like to thank all them for their special contribution and dedication to improving knowledge on this disease. The ultimate aim of this work is to improve the lives of our patients and thus any insight regarding future implementation of LB in a clinical setting is essential. We would also like to thank all those patients that have contributed selflessly to the advancement of this knowledge.

We hope that you enjoy reading this journals collection, with the wish that it increases the knowledge of those working in this area and that it may be also a good introduction for new researchers that have just discovered this exciting area.

Thanks for reading.
